# Cellular Debris in the Anterior Vitreous in Silent Eyes of Behçet Patients as a Clue of Asymptomatic Ocular Involvement

**DOI:** 10.1155/2013/398054

**Published:** 2013-10-03

**Authors:** Abdullah Kursat Cingu, Fatih Mehmet Türkcü, Bariş Yeniad, llknur Tugal-Tutkun

**Affiliations:** ^1^Department of Ophthalmology, Faculty of Medicine, Dicle University, 21280 Diyarbakir, Turkey; ^2^Department of Ophthalmology, Istanbul Faculty of Medicine, Istanbul University, 34104 Istanbul, Turkey

## Abstract

*Purpose*. To investigate if there is a prognostic implication of the presence of cellular debris in the anterior vitreous in patients with Behçet's disease (BD) without any ocular symptoms. *Methods*. One hundred and twenty eyes of 60 patients with BD were included in the study. The eyes were divided into two groups according to the presence of cellular debris in the anterior vitreous. The first group included 54 eyes which were cellular debris (+) (group A), and the second group included 66 eyes which were cellular debris (−) (group B). Fluorescein angiography (FA) was performed to all patients following routine ocular examination. Patients were called for the six monthly control visits to investigate possible new ocular involvement during followup. The Kaplan-Meier method was used to estimate survival curves. *Results*. Seven eyes (13%) in group A and four eyes (6.1%) in group B showed optic disc hyperfluorescence on FA (*P* = 0.2). None of the eyes with disc hyperflourescence on initial examination developed uveitis attacks in their followup. In Kaplan-Meier survival analysis there was a significant difference between the groups (group A 20.6% and group B 4.2%) by means of ocular involvement during their followup (log-rank = 6.85, *P* = 0.009). *Conclusions*. Presence of cellular debris in the anterior vitreous may have prognostic implications in patients with BD screened for ocular involvement.

## 1. Introduction

Uveitis occurs in 50–70% of patients with Behçet's disease (BD) typically within two years of disease onset in the majority of cases [1, 2]. The typical form of ocular involvement is bilateral panuveitis and retinal vasculitis. The course of the disease is characterized by the variable severity of recurrent symptomatic exacerbations of uveitis and spontaneous remission of the inflammatory signs [3]. Mild inflammatory episodes of anterior uveitis may go unnoticed by the patients as ciliary injection, pain or photophobia may be absent, and anterior chamber cells may resolve spontaneously [4]. Patients may also have asymptomatic posterior segment involvement early in the disease course. Fluorescein angiography (FA) has been reported to be a useful tool in the detection of asymptomatic ocular involvement [5, 6]. Laser flare photometry (LFP) is also a very useful tool in monitoring Behçet's uveitis [7]. In clinical practice it is usually used as an auxiliary method to show disease activity in the followup of uveitis patients.

Presence of vitreous cells on slit-lamp examination is accepted as eye involvement according to International Study Group (ISG) criteria for the diagnosis of BD [8]. However, in case of mild cellular debris in anterior vitreous without any other ocular signs and symptoms, diagnosis of ocular involvement may be controversial. 

The present study was conducted to investigate if there is a prognostic implication of having cellular debris in anterior vitreous in the development of obvious ocular involvement in the followup of patients with BD without any ocular symptoms. To our knowledge this is the first study which investigates the importance of cellular debris in anterior vitreous in patients with BD without any ocular symptoms.

## 2. Methods

The study included 120 eyes of 60 patients who were diagnosed as BD but had no history of ocular involvement and were found to have no ocular pathology or only cellular debris in the anterior vitreous on routine ocular examination at the uveitis services of two university hospitals between 2002 and 2013. Only patients who met the 1990 classification criteria of the ISG for BD were included in the study [8]. Patient informed consent was obtained from all participants in accordance with the Declaration of Helsinki, and ethics approval was obtained from Local Ethical Committee.

All patients were referrals from the dermatology or rheumatology clinics for initial ocular examination after they were diagnosed as BD. A complete ocular examination was performed, including visual acuity, slit-lamp biomicroscopy, tonometry, and indirect ophthalmoscopy. Patients who had a history of ocular involvement or who received any systemic treatment other than colchicum dispert and patients who had cells in the anterior chamber, posterior synechiae, cells or debris in the posterior vitreous, retinal vascular sheathing, retinal scars, or hyperemia or pallor of the optic disc were not included in the study. After a written informed consent was obtained, FA was performed on the day of initial visit. The angiograms were later evaluated by a masked observer. 

Patients were divided into two groups according to the presence of cellular debris in the anterior vitreous detected at the slit-lamp examination. The groups were defined as cellular debris (+) (group A) and cellular debris (−) (group B). Patients were followed up without any additional systemic or topical treatment unless they developed uveitis attacks later during their followup period.

*Statistical Analysis*. SPSS statistics software package version 15.0 for Windows (SPSS, Chicago, IL, USA) was used for the statistical analysis. Since the data did not follow Gaussian's distribution continuous variables were compared with Mann-Whitney *U* test. Categorical data were analyzed with the Fisher exact test. The Kaplan-Meier method was used to estimate survival curves of the patients followed up for at least 6 months. The log-rank test was used to perform comparisons of survival curves between the groups. A *P* value of less than 0.05 was considered statistically significant.

## 3. Results

**Figure 1:**
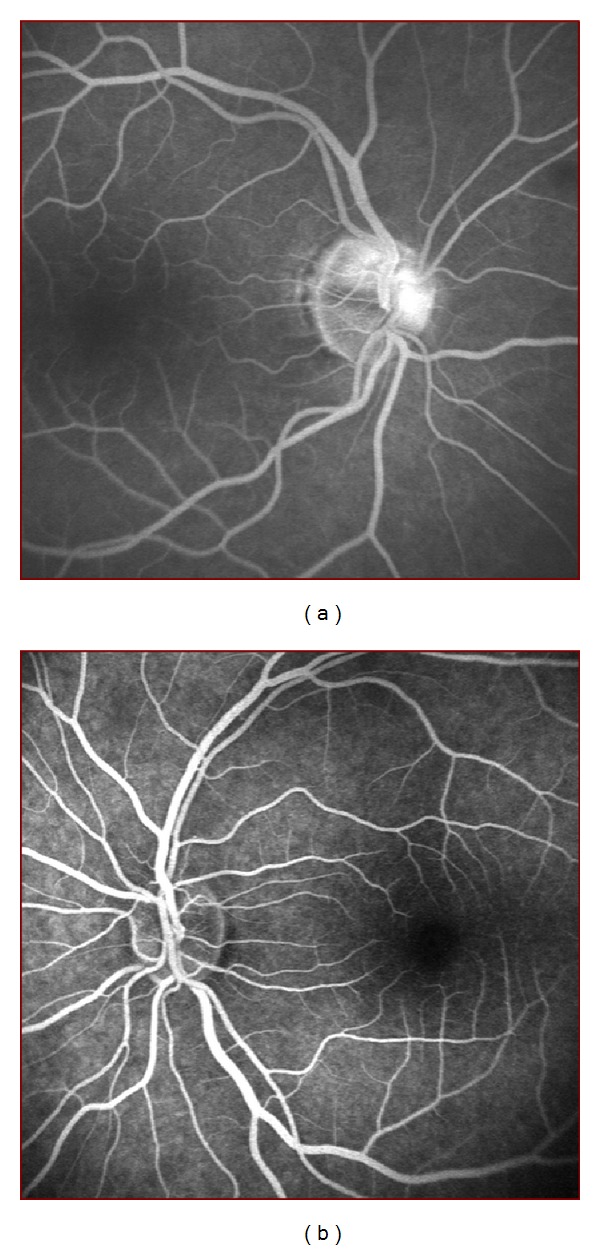
(a) FA images of a patient showing optic disc hyperfluorescence and (b) a normal FA image of another patient.

Of the 60 patients, 30 were males and 30 were females. The mean age was 27.8 ± 7.8. Ocular examination revealed no pathologic findings in 28 patients. Cellular debris was seen in the anterior vitreous bilaterally in 22 patients and unilaterally in 10 patients. Thus, there were 54 eyes in the cellular debris (+) group and 66 eyes in the cellular debris (−) group. Seven eyes (13%) in group A and four eyes (6.1%) in group B demonstrated optic disc hyperfluorescence (staining of the disc) on FA at initial examination. There was no statistically significant difference between the two groups in optic disc hyperfluorescence (*P* = 0.2).  Figure 1 shows FA images of two different patients, one having optic disc hyperfluorescence and the other being normal. There was no other angiographic abnormality in any eye in either group.

**Figure 2:**
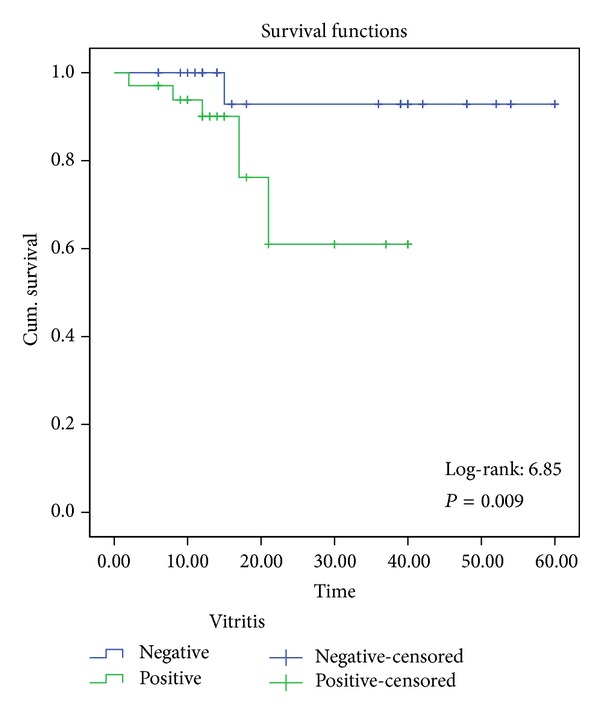
Survival functions of the groups according to ocular attack. Green line represents group A and blue line represents group B. A censored observation represents the end of their followup time for each patient. Steps at 2nd, 8th, 12th, 17th (binocular involvement), and 21st (binocular involvement) months in group A and 15th month (binocular involvement) in group B show individual ocular attacks. Also 60th and 40th months are the end of the last survived patients' followup period for group A and group B, respectively.

Forty-one patients were followed up for at least six months. Among 82 followed up eyes there were 34 eyes in group A and 48 eyes in group B.  Table 1 shows that the patients experienced uveitis attacks during their followup period. Kaplan-Meier survival analysis estimated the risk of having uveitis attack within 3 years for group A and group B as 20.6% (7 eyes) and 4.2% (2 eyes), respectively. The survival rates in terms of uveitis attacks were 35.7 ± 2.8 and 56.7 ± 2.1 in group A and group B, respectively (log-rank = 6.85, *P* = 0.009) (Figure 2). None of the uveitis attacks occurred in the eyes that had disc hyperfluorescence on initial examination.

## 4. Discussion

Fluorescein angiography has been an essential tool in both the diagnosis and followup of patients with Behçet's uveitis. It has also been reported to be a useful method in the early detection of ocular involvement [9, 10]. Dye leakage from the retinal vessels may be found during remission periods in the eyes with the history of posterior uveitis attacks [11]. In a previous study we found fluorescein leakage in 52 of 99 eyes (52.5%) during clinical remission period following a documented posterior uveitis attack [12]. Fluorescein leakage from retinal vessels may be seen in eyes without ophthalmoscopic signs of vasculitis. In one study it has been reported to be the only ocular finding that confirmed the dermatological diagnosis of BD in 6.3% of 300 patients [9]. However FA is an invasive intervention and has some complications [13–15].

Laser flare photometry (LFP) is also a very useful tool in monitoring Behçet's uveitis like FA. In a previous study from our clinic there was a strong correlation of flare values with FA leakage suggesting that LFP may be reliably used to quantify changes in the blood aqueous barrier functions in the followup of patients with BD [7]. In another study from our clinic the risk of a recurrent uveitis attack was found higher in patients with flare readings more than 6 photons/msec than in patients with lower flare readings [12]. This cut-off point would also be helpful in the differentiation of nonocular and ocular Behçet patients. But it does not give the risk of experiencing a uveitis attack for nonocular Behçet patients whom flare levels were very similar to those of normal population (3.5 photons/msec). The present study was designed before we had the LFP device so we could not be able to perform LFP to all patients.

We previously found that 35% of unilateral Behçet uveitis patients became bilateral at the end of the 3-year followup period [16]. However in the literature there is no study evaluating the risk of experiencing a uveitis attack in nonocular BD patients.

In this study we performed FA to all patients at initial examination, and we found optic disc hyperfluorescence as the only FA finding in 11 of 120 eyes (9.1%). Although this finding was more common in group A than in group B (13% versus 6.1%) it was not statistically significant. We performed Kaplan-Meier survival analysis to the patients and found the risk of having uveitis attack within 3 years 5 times higher in group A than in group B. There was no such risk for the eyes with previous disc hyperfluorescence, and none of the uveitis attacks occurred in these eyes. 

Subclinical ocular involvement might be detectable with fluorescein and/or (if available) indocyanine green angiography. Because most of the patients did not accept to be followed up with FA, in their followup visits, we perform only slit-lamp biomicroscopy and ophthalmoscopic examinations to the patients. So we might miss some subclinical ocular attacks in the followup period.

Cellular debris in the anterior vitreous may implicate a subclinical anterior uveitis, and in the current study we found that it has prognostic implications in patients with BD screened for ocular involvement. Additionally, periodic ocular examination is very important in all Behçet patients evenifthey are asymptomatic for ocular involvement, especially those with cellular debris in their anterior vitreous.

## Figures and Tables

**Table 1:** The patients experienced uveitis attack during their followup.

	Group	Age	Gender	Interval (mo)	Laterality of attack	Type of attack
Patient 1	B	28	Male	15	Bilateral	Posterior
Patient 2	A	25	Male	2	Unilateral	Anterior
Patient 3	A	19	Female	8	Unilateral	Anterior
Patient 4	A	23	Male	21	Bilateral	Anterior
Patient 5	A	20	Female	12	Unilateral	Anterior
Patient 6	A	24	Male	17	Bilateral	Anterior

## References

[1] Demiroglu H, Barista I, Dundar S (1997). Risk factor assessment and prognosis of eye involvement in Behçet’s disease in Turkey. *Ophthalmology*.

[2] Kural-Seyahi E, Fresko I, Seyahi N (2003). The long-term mortality and morbidity of Behçet syndrome: a 2-decade outcome survey of 387 patients followed at a dedicated center. *Medicine*.

[3] Tugal-Tutkun I, Onal S, Altan-Yaycioglu R, Huseyin Altunbas H, Urgancioglu M (2004). Uveitis in Behçet disease: an analysis of 880 patients. *American Journal of Ophthalmology*.

[4] Sakane T, Takeno M, Suzuki N, Inaba G (1999). Behçet’s disease. *The New England Journal of Medicine*.

[5] Gedik Ş, Akova YA, Yilmaz G, Bozbeyoğlu S (2005). Indocyanine green and fundus fluorescein angiographic findings in patients with active ocular Behçet’s disease. *Ocular Immunology and Inflammation*.

[6] Atmaca LS, Sonmez PA (2003). Fluorescein and indocyanine green angiography findings in Behçet’s disease. *British Journal of Ophthalmology*.

[7] Tugal-Tutkun I, Onal S, Altan-Yaycioglu R, Kir N, Urgancioglu M (2006). Neovascularization of the optic disc in Behçet’s disease. *Japanese Journal of Ophthalmology*.

[8] Wechsler B, Davatchi F, Mizushima Y (1990). Criteria for diagnosis of Behçet’s disease. *The Lancet*.

[9] Atmaca LS (1989). Fundus changes associated with Behçet’s disease. *Graefe’s Archive for Clinical and Experimental Ophthalmology*.

[10] Isik C, Yildirim C, Tatlipinar S, Yaylali V, Gungen S, Ozden S (2006). Klinik olarak okuler tutulumu olmayan Behçet hastalarında fundus floresein anjiyografi bulgulari (Fundus fluorescein angiography findings in Behçet’s patients without clinical ocular manifestations. *Türk Oftalmoloji Gazetesi*.

[11] Mishima S, Masuda K, Izawa Y (1979). The eighth Frederick H. Verhoeff lecture. Behçet’s disease in Japan: ophthalmologic aspects. *Transactions of the American Ophthalmological Society*.

[12] Tugal-Tutkun I, Cingü K, Kir N, Yeniad B, Urgancioglu M, Gül A (2008). Use of laser flare-cell photometry to quantify intraocular inflammation in patients with Behçet Uveitis. *Graefe’s Archive for Clinical and Experimental Ophthalmology*.

[13] Kwiterovich KA, Maguire MG, Murphy RP (1991). Frequency of adverse systemic reactions after fluorescein angiography: results of a prospective study. *Ophthalmology*.

[14] Stein MR, Parker CW (1971). Reactions following intravenous fluorescein. *American Journal of Ophthalmology*.

[15] Yannuzzi LA, Rohrer KT, Tindel LJ (1986). Fluorescein angiography complication survey. *Ophthalmology*.

[16] Cingu AK, Onal S, Urgancioglu M, Tugal-Tutkun I (2012). Comparison of presenting features and three-year disease course in Turkish patients with Behçet uveitis who presented in the early 1990s and the early 2000s. *Ocular Immunology and Inflammation*.

